# Supplementary material for: Bridging the Translational Gap in Chemotherapy-Induced Peripheral Neuropathy with iPSC-Based Modeling
Source: Cancers (Basel). 2022 Aug 15;14(16):3939. doi: 10.3390/cancers14163939 (PMC9406154; doi:10.3390/cancers14163939)
Supplement: Supplementary file 1 [file cancers-14-03939-s001.zip › Figure S1, Table S1_v2.pdf]

Supplementary Materials

Figure S1: Flow diagram showing the overview of the literature search

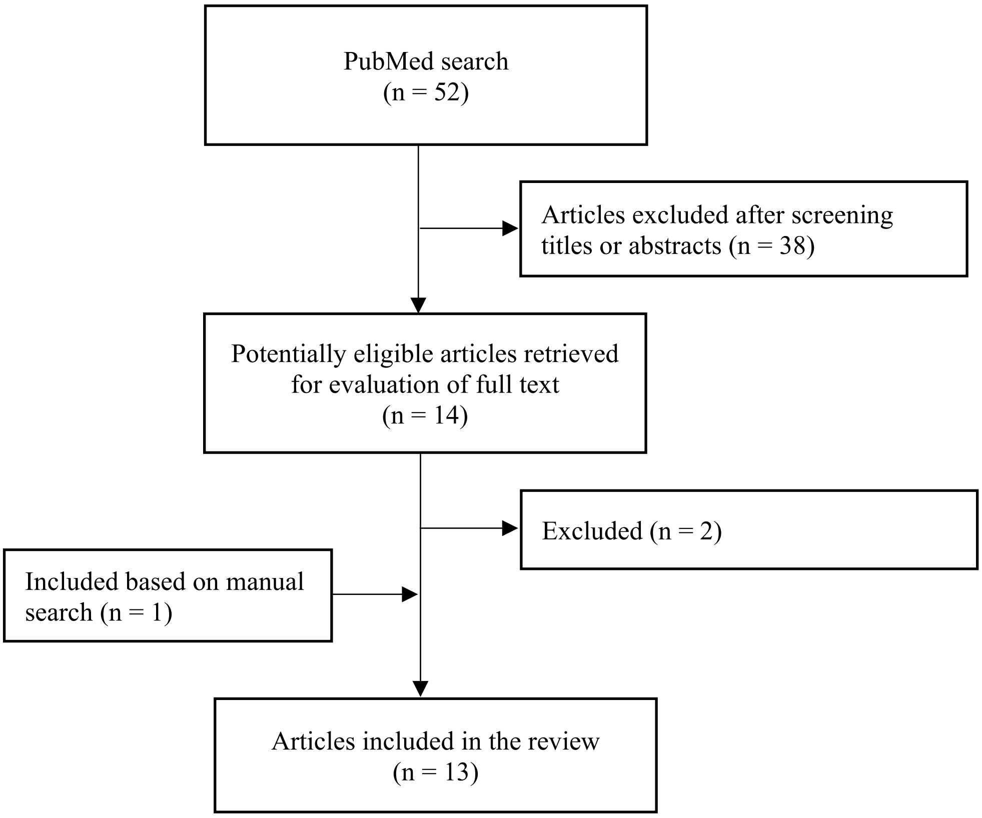

Table S1: PubMed search strategy

Terms for “chemotherapy-induced peripheral neuropathy”	("chemotherapy"[All Fields] OR "drug therapy"[MeSH Terms] OR ("drug"[All Fields] AND "therapy"[All Fields]) OR "drug therapy"[All Fields] OR "chemotherapies"[All Fields] OR "drug therapy"[MeSH Subheading] OR "chemotherapy"[All Fields]) OR ("neurodegenerative"[All Fields] OR "neurodegeneratives"[All Fields]) OR ("neurotoxic"[All Fields] OR "neurotoxical"[All Fields] OR "neurotoxicities"[All Fields] OR "neurotoxicity syndromes"[MeSH Terms] OR ("neurotoxicity"[All Fields] AND "syndromes"[All Fields]) OR "neurotoxicity syndromes"[All Fields] OR "neurotoxicity"[All Fields] OR "neurotoxics"[All Fields] OR

	"neurotoxity"[All Fields]) OR ("peripheral nervous system"[MeSH Terms] OR ("peripheral"[All Fields] AND "nervous"[All Fields] AND "system"[All Fields]) OR "peripheral nervous system"[All Fields]) (("chemotherapy-induced"[All Fields] AND ("peripheral nervous system diseases"[MeSH Terms] OR ("peripheral"[All Fields] AND "nervous"[All Fields] AND "system"[All Fields] AND "diseases"[All Fields]) OR "peripheral nervous system diseases"[All Fields] OR ("peripheral"[All Fields] AND "neuropathy"[All Fields]) OR "peripheral neuropathy"[All Fields])) OR ("chemotherapy-induced"[All Fields] AND ("neurotoxic"[All Fields] OR "neurotoxical"[All Fields] OR "neurotoxicities"[All Fields] OR "neurotoxicity syndromes"[MeSH Terms] OR ("neurotoxicity"[All Fields] AND "syndromes"[All Fields]) OR "neurotoxicity syndromes"[All Fields] OR "neurotoxicity"[All Fields] OR "neurotoxics"[All Fields] OR "neurotoxity"[All Fields])) OR ("chemotherapy-induced"[All Fields] AND ("toxic"[All Fields] OR "toxical"[All Fields] OR "toxically"[All Fields] OR "toxicant"[All Fields] OR "toxicant s"[All Fields] OR "toxicants"[All Fields] OR "toxicated"[All Fields] OR "toxication"[All Fields] OR "toxicities"[All Fields] OR "toxicity"[MeSH Subheading] OR "toxicity"[All Fields] OR "toxicity s"[All Fields] OR "toxics"[All Fields])) OR ("peripheral nervous system diseases"[MeSH Terms] OR ("peripheral"[All Fields] AND "nervous"[All Fields] AND "system"[All Fields] AND "diseases"[All Fields]) OR "peripheral nervous system diseases"[All Fields] OR ("peripheral"[All Fields] AND "neuropathy"[All Fields]) OR "peripheral neuropathy"[All Fields]))
Terms for "induced pluripotent stem cells"	("induced pluripotent stem cells"[MeSH Terms] OR ("induced"[All Fields] AND "pluripotent"[All Fields] AND "stem"[All Fields] AND "cells"[All Fields]) OR "induced pluripotent stem cells"[All Fields] OR ("human"[All Fields] AND "induced"[All Fields] AND "pluripotent"[All Fields] AND "stem"[All Fields] AND "cell"[All Fields]) OR "human induced pluripotent stem cell"[All Fields])
Terms for "PNS cell types"	("neuron s"[All Fields] OR "neuronal"[All Fields] OR "neuronally"[All Fields] OR "neuronals"[All Fields] OR "neurone s"[All Fields] OR

	"neurones"[All Fields] OR "neuronic"[All Fields] OR "neurons"[MeSH Terms] OR "neurons"[All Fields] OR "neuron"[All Fields] OR "neurone"[All Fields] OR ("neural"[All Fields] OR "neuralization"[All Fields] OR "neuralize"[All Fields] OR "neuralized"[All Fields] OR "neuralizes"[All Fields] OR "neuralizing"[All Fields] OR "neurally"[All Fields]) OR "neuronal"[All Fields] OR "neuronally"[All Fields] OR ("nerve"[All Fields] OR "nerve s"[All Fields] OR "nerved"[All Fields] OR "nerves"[All Fields]) OR ("schwann"[All Fields] OR "schwann s"[All Fields]) OR ("satellite"[All Fields])
